# A disproportionality analysis of adverse event reports for givinostat in the FDA adverse event reporting system (FAERS) database

**DOI:** 10.3389/fphar.2026.1954821

**Published:** 2026-10-21

**Authors:** Lin Chen, Hanyu Zhang, Yunlong Wang, Xianhu Feng, Yi Hou, Qiang Su

**Affiliations:** 1 Nanchong Key Laboratory of Individualized Drug Therapy, Department of Pharmacy, Beijing Anzhen Nanchong Hospital Capital Medical University & Nanchong Central Hospital, The Second Clinical Medical College, North Sichuan Medical College, Nanchong, Sichuan, China; 2 West China of Hospital, Sichuan University, Chengdu, Sichuan, China

**Keywords:** Duchenne muscular dystrophy, FAERS, givinostat, pharmacovigilance, thrombocytopenia, safety signal

## Abstract

**Background:**

Givinostat was recently approved for Duchenne muscular dystrophy (DMD), but its real-world safety profile remains incompletely characterized. We evaluated givinostat-associated safety signals by data mining of the FDA Adverse Event Reporting System (FAERS) database.

**Methods:**

Reporting Odds Ratio (ROR), Proportional Reporting Ratio (PRR), Bayesian Confidence Propagation Neural Network (BCPNN), and Multi-item Gamma Poisson Shrinker (MGPS) were applied to 321 reports with givinostat as the primary suspect (PS) drug from Q1 2024 to Q1 2026. A likelihood ratio test (LRT) with Benjamini–Hochberg (BH) correction further assessed whether specific adverse events (AEs) were disproportionately associated with serious outcomes. Serious *versus* non-serious cases were compared by logistic regression and univariate tests.

**Results:**

At the system organ class (SOC) level, investigations (ROR 5.54), gastrointestinal disorders (ROR 4.09), and blood and lymphatic system disorders (ROR 3.43) showed the strongest signals. For the preferred term (PT) level, blood triglycerides exhibited the strongest signal (ROR 341.3), while platelet count decreased was the most frequently reported (*n* = 96, 29.9%). After BH adjustment, platelet count decreased was the only PT with a significantly lower serious proportion than the overall baseline (14.58% vs. 30.07%, adjusted *p* = 0.024). Time-to-Onset (TTO) Analysis showed that diarrhoea typically occurred within the first week, whereas platelet count decreased and thrombocytopenia had delayed median onset (15.5 days and 17 days, respectively).

**Conclusion:**

Our study identified thrombocytopenia and hypertriglyceridemia as the most prominent safety signals for givinostat, with platelet count decreased showing a statistically significant difference in serious proportion and a lower serious proportion than the overall baseline. These findings provide evidence-based targets for structured hematological and metabolic monitoring, particularly during the early treatment phase, to support risk management in DMD patients receiving givinostat.

## 1 Introduction

Duchenne muscular dystrophy (DMD) is a severe, X-linked recessive neuromuscular disorder caused by mutations in the DMD gene, leading to the absence of functional dystrophin (Roberts et al., 2023). This deficiency results in progressive muscle degeneration, loss of ambulation by adolescence, and ultimately, cardiorespiratory failure in early adulthood (Mendell et al., 2025; Jones, 2019). Currently, standard of care primarily relies on chronic corticosteroid therapy, which offers modest functional benefits but is accompanied by substantial long-term adverse effects, including weight gain, behavioral changes, and osteoporosis (Markati et al., 2022). Although novel genetic therapies, such as exon-skipping antisense oligonucleotides and micro-dystrophin gene transfer, have shown promise, their efficacy is limited to specific mutation subtypes and remains constrained by immunogenicity and delivery challenges (Wang et al., 2024). Consequently, there is an urgent, unmet clinical need for disease-modifying agents that can be broadly applied across the DMD patient population.

Givinostat, a pan-histone deacetylase (HDAC) inhibitor, has recently emerged as a compelling therapeutic candidate for DMD. HDAC inhibition has been shown to counteract the chronic inflammatory state, reduce muscle fibrosis, and promote muscle regeneration in preclinical mdx mouse models (Aartsma-Rus, 2025). Beyond its anti-inflammatory and anti-fibrotic effects, givinostat acts as a metabolic remodeler by inhibiting deacetylation of the PGC-1α promoter, thereby sustaining mitochondrial biogenesis and preserving oxidative fiber-type switching. This is particularly relevant in DMD, where two temporally distinct phases of mitochondrial damage have been described which are early mitochondrial mass depletion followed by accumulation of dysfunctional mitochondria (Giovarelli et al., 2021). Importantly, a pivotal Phase 3 randomized controlled trial (EPIDYS) demonstrated that givinostat significantly slowed the decline in motor function compared to placebo, leading to its recent conditional approval or expanded access in several regions (Mercuri et al., 2024). While the efficacy profile appears encouraging, the systemic nature of HDAC inhibition raises substantial safety concerns. Notably, Class I HDAC inhibitors are well-documented to interfere with megakaryocyte differentiation and platelet shedding, with thrombocytopenia emerging as a dose-limiting toxicity in earlier oncological trials (Marks and Breslow, 2007). Additionally, gastrointestinal intolerability has been reported, yet the comprehensive safety spectrum of givinostat in patients with DMD remains inadequately defined in real-world settings (Mercuri et al., 2024).

In this study, we performed a disproportionality analysis using the FAERS database to quantitatively detect givinostat-associated safety signals from Q1 2024 to Q1 2026. To comprehensively identify potential signals, we applied four disproportionality methods (ROR, PRR, BCPNN, and MGPS). Furthermore, we conducted subgroup analyses stratified by age, weight, and TTO, as well as a likelihood ratio test with BH correction to formally assess whether specific AEs were disproportionately associated with serious outcomes. Through this multi-dimensional analytical approach, we aimed to provide robust pharmacovigilance evidence to inform risk-management strategies for DMD patients receiving givinostat.

## 2 Methodology

### 2.1 Data sources

The study is designed as an observational, retrospective disproportionality analysis, a validated pharmacovigilance approach to assess whether an association is likely to exist between a drug and target AEs (Shu et al., 2022). This study utilized data from FAERS from Q1 2024 to Q1 2026, capturing the post-launch period since givinostat’s approval in 2024. The FAERS data were downloaded from the FAERS Quarterly Data Extract Files, available at https://fis.fda.gov/extensions/FPD-QDE-FAERS/FPD-QDE-FAERS.html. According to the FDA approval time of givinostat, all reports recorded in FAERS covering the period from the first quarter (Q1) of 2024–2026 Q1, were included in our analysis.

### 2.2 Data extraction and descriptive analysis

The FAERS database consisted of seven data files: patient demographic information (DEMO), drug information (DRUG), adverse event information (REAC), patient outcome information (OUTC), report source information (RPSR), drug therapy date information (THER), and drug indication (INDI) (Dai et al., 2025). In addition, the cases deleted by FDA or manufacturers for various reasons including combining cases, were shown in Deleted files. All data were imported and processed using R software (version 4.4.1), and the deduplication process was performed before statistical analysis. The primary ID was the primary link field (primary key) between different data files, and the case ID was chosen as the key filter in our study to remove duplicate records (Chen et al., 2024). We identified cases using generic name (givinostat and givinostat hydrochloride in drugname and prod_ai columns) and trade name (Duvyzat in drugname column) in the DRUG file, and chose the role_cod as PS. AEs in FAERS are coded using the PT from standardized Medical Dictionary for Regulatory Activities (MedDRA), which contains 27 SOCs. Further, a PT can be linked to more than one SOC in MedDRA. Subsequently, the clinical characteristics of reports were described in detail, if the data were available, including gender, age, weight, reporting area, indications, outcomes and reporters, *etc.* Notably, serious adverse events (SAEs) were defined using the outcome codes DE (death), HO (hospitalization), LT (life-threatening), or OT (other serious outcomes), consistent with previous FAERS studies (Sonawane et al., 2018). Because FAERS does not explicitly code non-serious events, reports without these serious outcome codes were operationally classified as non-serious for comparative analyses. However, this category should be interpreted as “no documented serious outcome” rather than confirmed non-serious events. Of note, total serious outcomes may exceed the total number of reports because some cases list more than one serious outcome. A flow diagram including the multi-step process of data extraction, processing, and analysis was shown in Figure 1.

**FIGURE 1:**
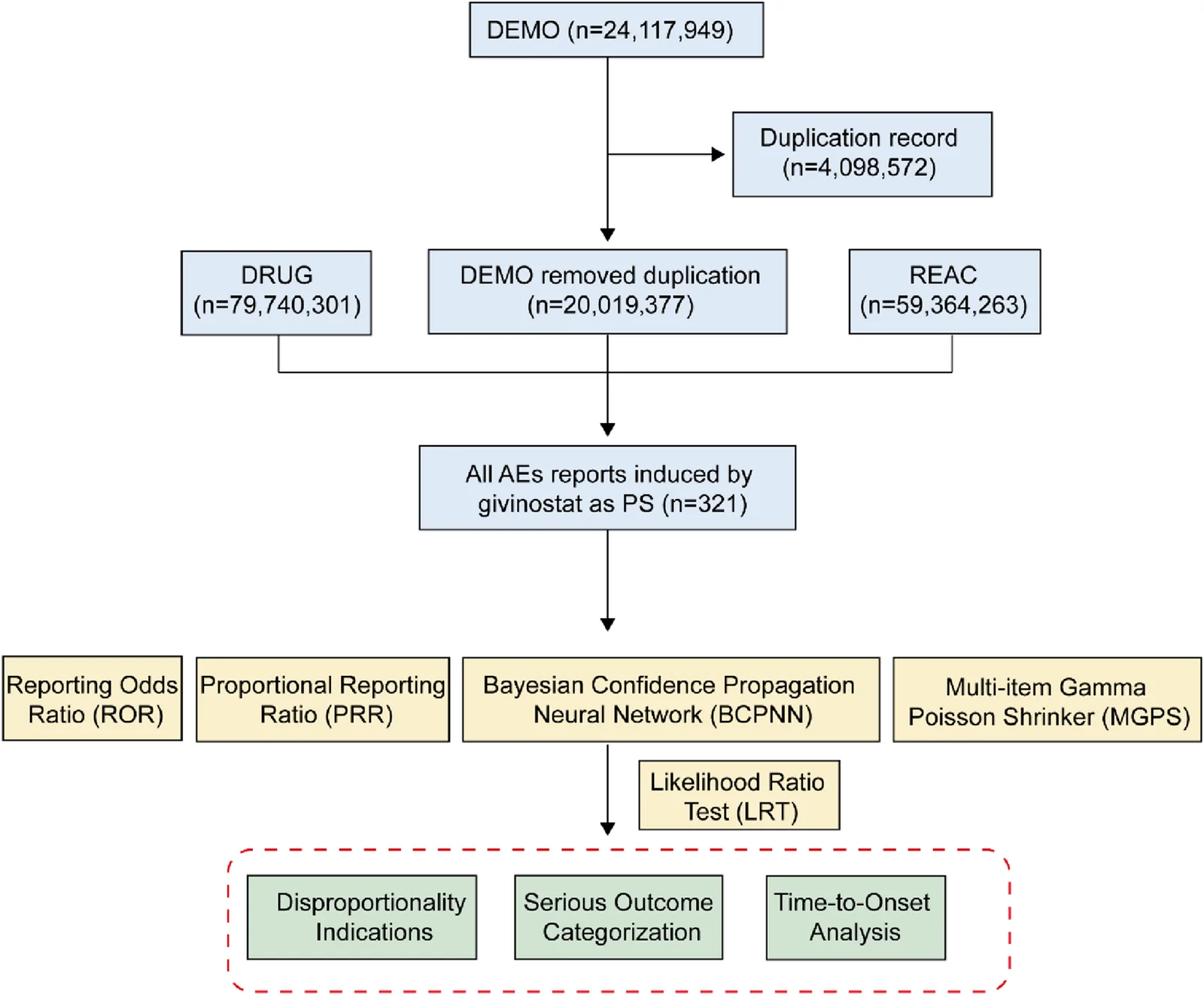
Flowchart of the data selection process from the FAERS database (Q1 2024 – Q1 2026).

In the FAERS/MedDRA hierarchy, laboratory abnormalities and clinical diagnoses are coded as distinct PTs. “Platelet count decreased” (PT, primary SOC: investigations) denotes a laboratory finding of reduced platelet count and does not necessarily imply a clinical diagnosis. “Thrombocytopenia” (PT, primary SOC: blood and lymphatic system disorders) denotes a clinician-coded clinical diagnosis. These two PTs were analyzed independently and were not collapsed into a composite endpoint. When both PTs appeared in the same report, the report was retained in each PT-specific analysis, and the overlap was quantified. Seriousness was defined solely by OUTC outcome codes (DE, HO, LT, or OT) and was not inferred from the PT itself. In the text, “thrombocytopenia” is used only for the PT “Thrombocytopenia”; when both entities are intended, the term “platelet-related adverse events” is used.

### 2.3 Statistical analysis

Given the inherent limitations of spontaneous reporting data, this analysis is hypothesis-generating and does not confirm causality. To detect safety signals, we constructed a 2 × 2 contingency table (Table 1) and applied four disproportionality methods: ROR, PRR, BCPNN, and MGPS. The formulas and thresholds for each method are detailed in Table 2. ROR and PRR provide frequentist estimate of association strength; BCPNN and MGPS, as Bayesian shrinkage estimators, offer enhanced stability for sparse data. A signal was considered positive if it met the criteria for at least three of the four methods. To formally assess host-factor-related reporting bias, we further adopted the LRT framework proposed by Lu et al. (2023), comparing each PT against all other PTs within givinostat reports across levels of age, weight, TTO, and outcome seriousness (Lu et al., 2023). Given the limited case numbers (fewer than 5 SAE events) in certain age- and weight-specific subgroups, we consolidated these variables for multivariate analysis. Accordingly, age was dichotomized as pediatric (<18 years) *versus* adult (≥18 years), and weight was dichotomized as ≤45 kg *versus* >45 kg, based on clinical cutoffs. Corticosteroid use was coded as a binary variable (Yes vs. No). These variables were included in univariable and multivariable logistic regression models with SAE as the dependent variable. The 45 kg cutoff was selected based on commonly used weight stratification criteria in previous drug safety studies, with specific reference to the weight-based dosing thresholds used in DMD therapies (e.g., 40 kg for vamorolone). This value was conservatively adjusted upward to account for the lower lean body mass typical of this patient population. The BH procedure was applied to control the false discovery rate at 0.05. For TTO Analysis, we calculated the interval from the reported start date of givinostat to the date of adverse event onset. Only reports with complete and plausible dates (event date ≥ start date) were retained. Median TTO with interquartile range (IQR) was calculated for key PTs that showed significant disproportionality signals. Comparisons of TTO between serious and non-serious reports were performed using the Wilcoxon rank-sum test. In an exploratory active-comparator analysis, we compared selected disproportionality signals between givinostat and other therapies used in DMD, including prednisone, deflazacort, and vamorolone. These agents were identified from the DRUG file by generic and trade names. RORs were calculated using the same 2 × 2 framework. Because the number of reports for some comparators was small, RORs were not calculated when fewer than three reports were available and are marked as “–”. These comparisons are exploratory and should not be interpreted as head-to-head risk estimates.

**TABLE 1:** The 2 × 2 contingency table for disproportionality analysis.

Adverse event category	Target drug	Other drugs	Total
AEs of interest	a	b	a+b
Other AEs	c	d	c + d
Total	a+c	b + d	a+b + c + d

The variables are defined as follows: a, the number of reports for the target drug and the adverse event of interest; b, the number of reports for other drugs and the adverse event of interest; c, the number of reports for the target drug and all other AEs; d, the number of reports for all other drugs and all other AEs.

**TABLE 2:** Signal detection algorithms, formulas, and established thresholds used in the analysis.

Method	Formula	Threshold
PRR	PRR = (a/(a+b))/(c/(c + d))	PRR ≥2 and lower CI > 1
ROR	ROR = (a/c)/(b/d)	a ≥3 and 95% CI > 1
BCPNN	IC = log2 ((p (x,y))/(p(x)p(y)))	IC_025_ > 0
MGPS	EBGM = a/[(a+b) (a+c)/(a+b + c + d)]	EBGM_05_ > 2

IC, information component; IC_025_, the lower bound of the 95% confidence interval for IC; EBGM, empirical bayes geometric mean; EBGM_05_, the lower bound of the 95% confidence interval for EBGM; CI, Confidence Interval. the variables a, b, c, and d are defined in Table 1. The EBGM, formula shown is a simplified representation; the actual calculation is based on a Gamma-Poisson model.

## 3 Results

### 3.1 Demographic information on the AE reports

According to the FAERS database, a total of 24, 117, 949 AE reports were recorded between Q1 2024 and Q1 2026. After deduplication, 321 AE reports involving givinostat were identified. Reporting peaked in 2024 (265, 82.55%), with a marked decline in 2025 (36, 11.21%) and the first quarter of 2026 (20, 6.23%). The United States accounted for the vast majority of reports (306, 95.3%), indicating a primary reliance on the North American market for post-marketing surveillance of this drug. In terms of demographic characteristics, male patients predominated (308, 96.0%), with only one female case (0.3%). This male predominance is consistent with the X-linked recessive inheritance of DMD, the only indication for givinostat. The rare female cases may be explained by extremely skewed X-inactivation, Turner syndrome (45, XO), or X-autosome translocations leading to abnormal phenotypic expression. The age distribution was broad, with the highest proportion in adolescents aged 12–17 years (91, 28.3%), followed by children aged 2–11 years and adults aged 18–64 years (79 each, 24.6%). The notable adult proportion may reflect extended survival of DMD patients on continued therapy, or possible off-label use, which warrants further verification. However, the missing age rate was 22.4%, which limits the robustness of age-stratified subgroup analyses. Regarding reporting sources and outcomes, physicians were the primary reporters (86, 26.8%), and along with other health professionals (5.0%), suggesting overall good data credibility. Among documented outcomes, outcome data were missing for 257 cases (80.1%), while 8 DEs (2.5%), 37 OT (11.5%) and 19 HOs (5.9%) were recorded. These serious events necessitate ongoing pharmacovigilance monitoring. For route of administration, oral use was reported in 4.98% of cases, consistent with systemic DMD therapy, but the route variable was missing in 91.28% of reports, severely restricting any meaningful exposure-response evaluation (Table 3).

**TABLE 3:** Demographic and clinical characteristics of adverse event reports for givinostat in the FAERS database (2024–2026).

Variable	Total
Year	
2024	265 (82.55%)
2025	36 (11.21%)
2026	20 (6.23%)
Sex	
Missing	12 (3.7%)
Male	308 (96.0%)
Female	1 (0.3%)
Age	
12–17	91 (28.3%)
18–64	79 (24.6%)
2–11	79 (24.6%)
Missing	72 (22.4%)
Reporter type	
Consumer	8 (2.5%)
Health professional	16 (5.0%)
Physician	86 (26.8%)
Missing	203 (63.2%)
Pharmacist	8 (2.5%)
Outcome	
DE	8 (2.5%)
HO	19 (5.9%)
Missing	257 (80.1%)
OT	37 (11.5%)
Reporter country	
Unknown source	2 (0.6%)
France	2 (0.6%)
Germany	4 (1.2%)
United Kingdom	7 (2.2%)
United States	306 (95.3%)
Route	
Missing	293 (91.28%)
Oral	16 (4.98%)
Topical	7 (2.18%)
Unknown	5 (1.56%)

### 3.2 Signal detection at the SOC level

To characterize the overall safety profile of givinostat, we conducted disproportionality analysis at the SOC level, with results summarized in Table 4. In terms of signal strength, there were significant differences in the ROR values among the various SOCs. Among them, the investigations had the highest signal strength (ROR 5.54, 95% CI 4.71–6.53; IC 2.13, IC_025_ 1.87), followed by the blood and lymphatic system disorders (ROR 3.43, 95% CI 2.51–4.71; IC 1.72, IC_025_ 1.19) and the gastrointestinal disorders (ROR 4.09, 95% CI 3.48–4.80; IC 1.70, IC_025_ 1.45). The IC_025_ values of the above three SOCs were all greater than 0, indicating that the positive signal threshold was reached. Notably, although the number of reports for gastrointestinal disorders was the highest, the ROR signal strength of the investigations was higher (ROR 5.54 vs. 4.09), indicating that abnormal laboratory tests were the most frequently reported event category in the FAERS database. In contrast, the ROR values of the following SOCs were below one and IC_025_ was below 0, including general disorders and administration site conditions (ROR 0.43), nervous system disorders (ROR 0.42), infections and infestations (ROR 0.48), and musculoskeletal and connective tissue disorders (ROR 0.59), suggesting that the reporting frequency of these categories of events in this drug was lower than the background frequency of the database.

**TABLE 4:** Signal detection analysis for givinostat at the SOC level.

SOC	Reports	ROR (95% CI)	PRR (95% CI)	*χ* ^*2*^	IC (IC_025_)	EBGM (EBGM_05_)
Investigations	193	5.54 (4.71–6.53)	4.37 (3.87–4.93)	532.52	2.13 (1.87)	4.37 (3.71)
Gastrointestinal disorders	203	4.09 (3.48–4.80)	3.25 (2.89–3.65)	344.39	1.70 (1.45)	3.25 (2.76)
Injury, poisoning and procedural complications	71	0.99 (0.77–1.26)	0.99 (0.79–1.23)	0.01	−0.02 (−0.38)	0.99 (0.77)
General disorders and administration site conditions	61	0.43 (0.33–0.56)	0.48 (0.38–0.61)	41.88	−1.06 (−1.44)	0.48 (0.37)
Blood and lymphatic system disorders	41	3.43 (2.51–4.71)	3.3 (2.45–4.44)	66.85	1.72 (1.19)	3.30 (2.41)
Skin and subcutaneous tissue disorders	32	0.79 (0.55–1.13)	0.8 (0.57–1.12)	1.71	−0.32 (−0.83)	0.80 (0.56)
Nervous system disorders	27	0.42 (0.29–0.62)	0.44 (0.30–0.64)	20.78	−1.18 (−1.71)	0.44 (0.30)
Musculoskeletal and connective tissue disorders	23	0.59 (0.39–0.90)	0.61 (0.41–0.91)	6.22	−0.72 (−1.30)	0.61 (0.40)
Infections and infestations	19	0.48 (0.3–0.75)	0.49 (0.31–0.76)	10.65	−1.03 (−1.65)	0.49 (0.31)
Respiratory, thoracic and mediastinal disorders	17	0.48 (0.29–0.77)	0.49 (0.31–0.78)	9.52	−1.03 (−1.68)	0.49 (0.30)
Cardiac disorders	16	0.85 (0.52–1.39)	0.85 (0.52–1.38)	0.44	−0.24 (−0.93)	0.85 (0.52)
Metabolism and nutrition disorders	12	0.76 (0.43–1.35)	0.77 (0.44–1.34)	0.88	−0.39 (−1.17)	0.77 (0.43)
Renal and urinary disorders	7	0.52 (0.25–1.10)	0.53 (0.25–1.10)	3.00	−0.92 (−1.86)	0.53 (0.25)
Psychiatric disorders	5	0.12 (0.05–0.28)	0.12 (0.05–0.29)	33.20	−3.03 (−3.98)	0.12 (0.05)
Vascular disorders	5	0.32 (0.13–0.76)	0.32 (0.13–0.77)	7.29	−1.64 (−2.65)	0.32 (0.13)
Surgical and medical procedures	4	0.39 (0.14–1.03)	0.39 (0.15–1.04)	3.88	−1.36 (−2.47)	0.39 (0.15)
Ear and labyrinth disorders	2	0.64 (0.16–2.56)	0.64 (0.16–2.55)	0.41	−0.65 (−2.13)	0.64 (0.16)
Immune system disorders	2	0.24 (0.06–0.97)	0.24 (0.06–0.98)	4.72	−2.03 (−3.28)	0.24 (0.06)
Hepatobiliary disorders	1	0.15 (0.02–1.05)	0.15 (0.02–1.06)	4.90	−2.75 (−3.99)	0.15 (0.02)

### 3.3 Signal mining at the PT level

At the Preferred Term (PT) level, the top five terms in terms of reporting frequency are as follows: platelet count decreased (*n* = 96, ROR 91.7, 95% CI 73.97–113.68), diarrhoea (*n* = 62, ROR 8.29, 95% CI 6.39–10.75), blood triglycerides increased (*n* = 47, ROR 341.3, 95% CI 253.18–460.10), abdominal discomfort (*n* = 31, ROR 15.96, 95% CI 11.14–22.87), and thrombocytopenia (*n* = 30, ROR 23.76, 95% CI 16.48–34.24). According to the classification of disproportionate reporting signals, hematological toxicity was mainly manifested as platelet count decreased (*n* = 96) and thrombocytopenia (*n* = 30); lipid metabolism abnormalities were mainly reflected as blood triglycerides increased; and the bleeding tendency was represented by the contusion signal. Furthermore, at the PT level, ‘product dose omission issue’ and ‘off label use’ were also detected as non-safety-related signals. These were determined to be reporting biases at the operational or recording level and did not involve the pharmacological risks of the drug itself. They have been excluded from the final analysis.

To evaluate the consistency and robustness of signal detection across different methodologies, we analyzed the overlap of potential safety signals detected by the four algorithms (ROR, PRR, BCPNN, and MGPS) at the PT level (Figure 2). A total of 20 signals (33.9%) were concurrently identified by all four methods, representing highly consistent and robust safety signals, including key hematologic events (platelet count decreased, thrombocytopenia), gastrointestinal adverse effects (diarrhoea, nausea), and metabolic disturbances (blood triglycerides increased). Additionally, MGPS uniquely identified 33 signals (55.9%), reflecting its superior sensitivity in detecting rare or low-frequency events in sparse post-marketing data (*n* = 321).

**FIGURE 2:**
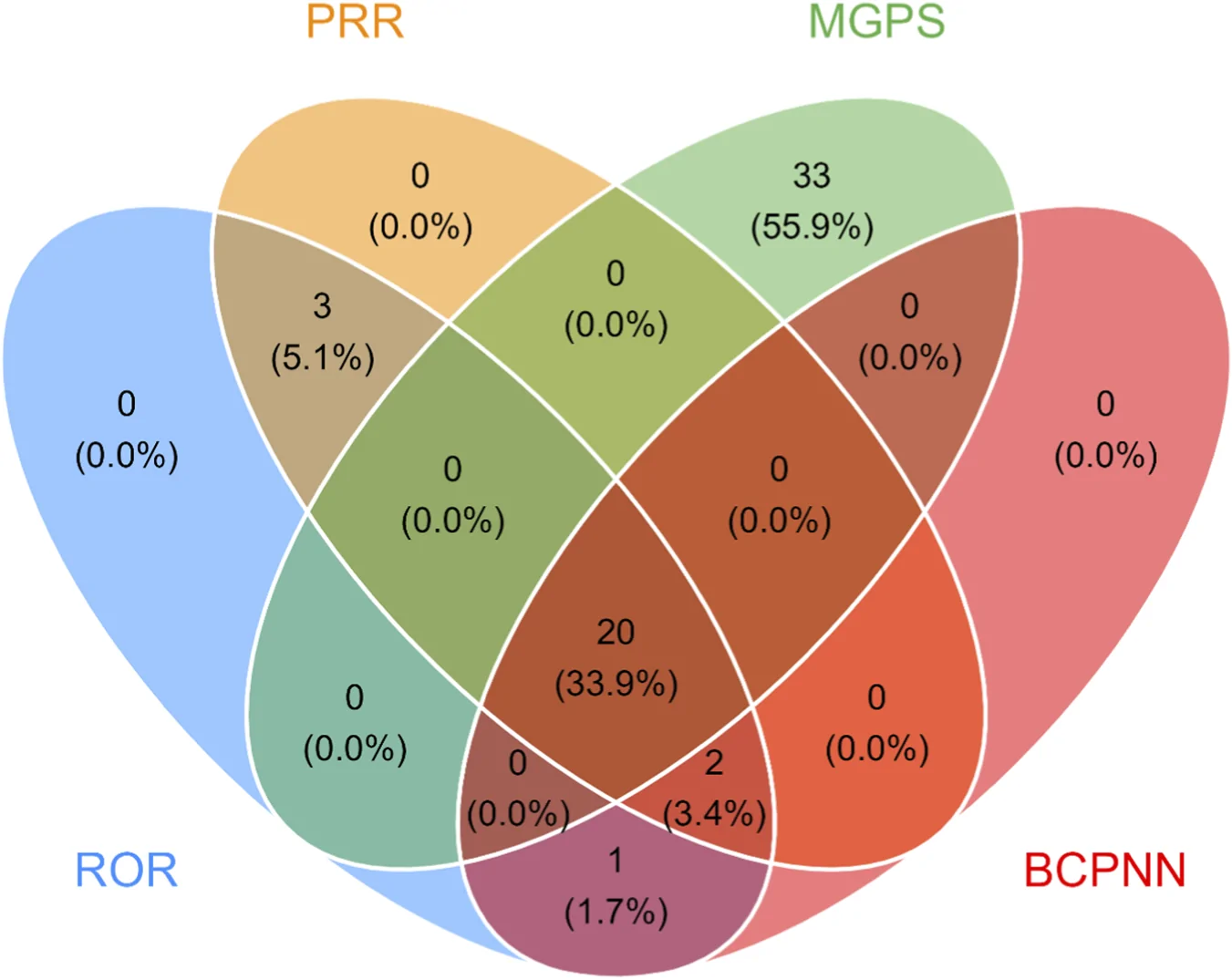
Venn diagram showing the overlap of safety signals detected by four disproportionality methods (ROR, PRR, BCPNN, and MGPS) at the Preferred Term level.

### 3.4 Frequency distribution of PT-level AEs

In terms of reporting frequency (Figure 3), the most frequently reported PT was platelet count decreased (29.91%), followed by diarrhoea (19.31%), blood triglycerides increased (14.64%), abdominal discomfort (9.66%), thrombocytopenia (9.35%), nausea (8.41%), and vomiting (7.17%). All high-frequency PTs were derived from SOC categories with positive disproportionality signals (IC_025_ > 0), including investigations, gastrointestinal disorders, and blood and lymphatic system disorders, reinforcing the cross-level concordance of these signals. Notably, blood triglycerides increased ranked third in reporting frequency (14.64%) but exhibited the highest ROR among all PTs (ROR 341.3, 95% CI 253.18–460.10). However, the decrease in platelet count, which was the most frequently reported PT indicator, did not show the strongest signal intensity. Additionally, events suggestive of bleeding tendency, including contusion (4.36%) and epistaxis (2.81%), and constitutional symptoms such as pyrexia (3.43%) and fatigue (1.87%), were reported at lower frequencies, consistent with the negative disproportionality signals observed at the SOC level for systemic and administration site conditions.

**FIGURE 3:**
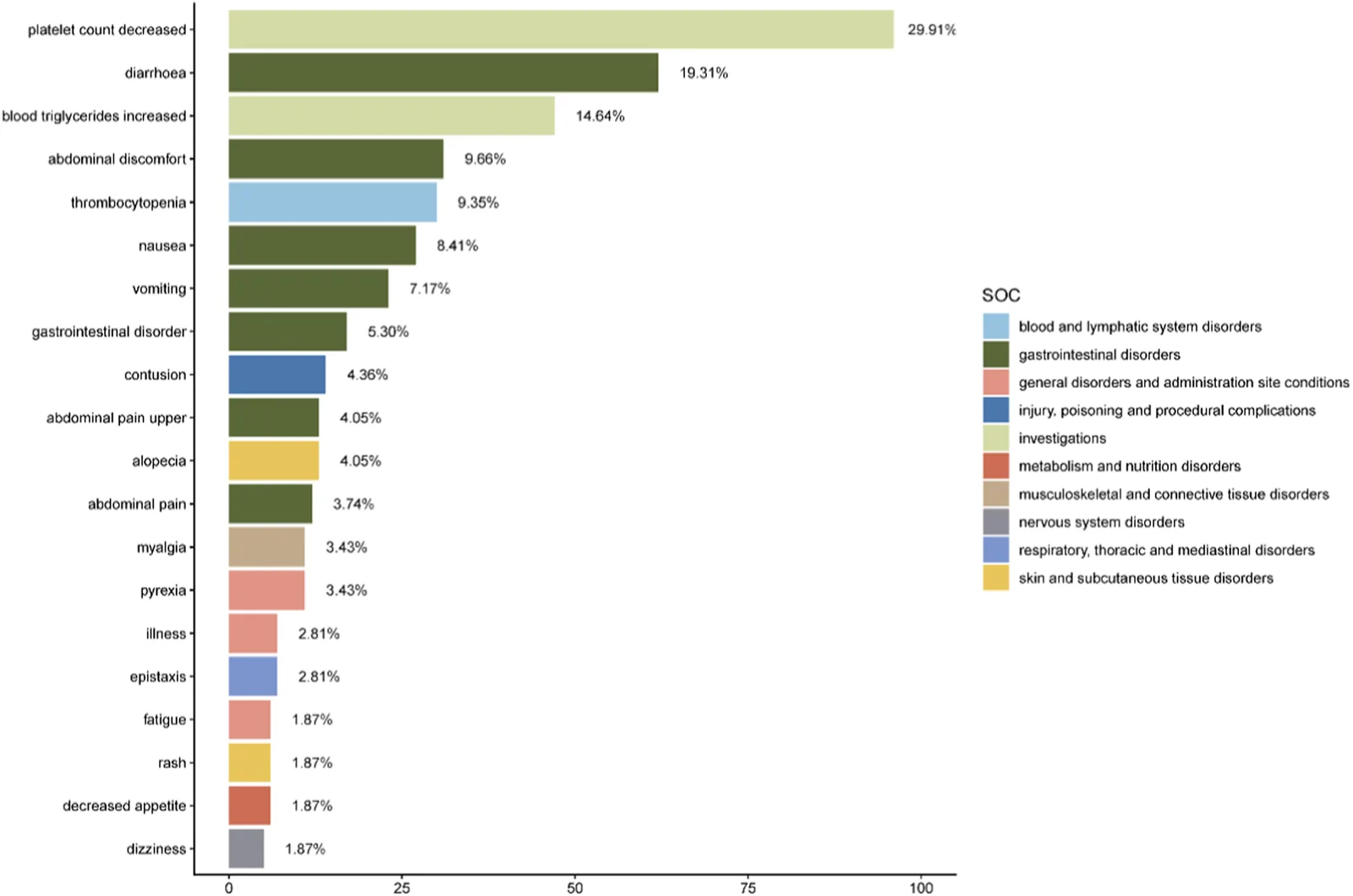
Frequency distribution of the top 20 most frequently reported PTs for givinostat.

As shown in Figure 4, platelet count decreased and blood triglycerides increased were situated at the extreme upper-right region, exhibiting the highest -log (*p*_adjust_) values and prominently elevated logRORs. The larger and darker point sizes for these terms reflect their relatively higher case counts. Concurrently, thrombocytopenia was positioned similarly high on the y-axis, further corroborating a robust hematological toxicity signal, particularly involving platelet-related abnormalities. In the mid-upper quadrant, abdominal discomfort and diarrhoea demonstrated clear statistical significance with positive logRORs, and diarrhoea showed a larger point size, indicating a higher reporting frequency. Additionally, terms such as petechiae, gastrointestinal disorder, and contusion displayed moderately elevated logRORs, consistent with a bleeding tendency secondary to platelet dysfunction, though their statistical significance and case numbers were slightly inferior to the top-ranked signals. Conversely, PTs clustered near the horizontal dashed line (significance threshold) or around the vertical line of null effect (logROR = 0), including headache, dizziness, fatigue, and rash, exhibited low -log (*p*_adjust_) values and logRORs approaching or below zero. This indicates that their reporting frequencies did not substantially exceed the background baseline of the FAERS database. Hematological toxicity (thrombocytopenia) and metabolic disturbance (hypertriglyceridemia) emerged as the most prominent signals, followed by gastrointestinal events. Notably, the analysis confirmed that several common constitutional symptoms were not disproportionately reported.

**FIGURE 4:**
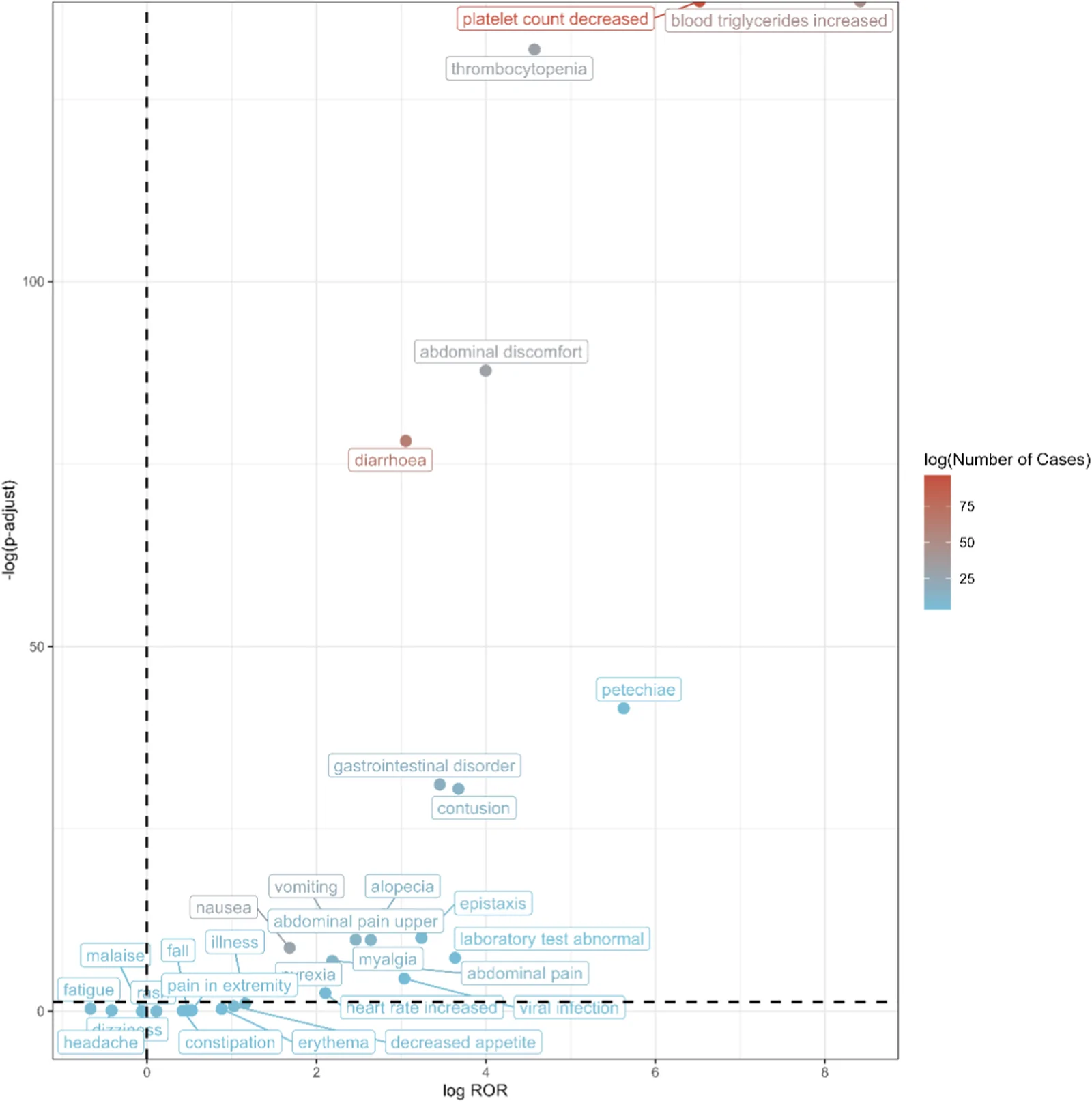
Volcano plot of logROR *versus* -log (adjusted *p*-value) for all reported PTs. The horizontal dashed line indicates the significance threshold (adjusted *p* < 0.05); the vertical dashed line indicates logROR = 0. Point size and color intensity reflect case counts.

As shown in Figure 5, the AEs related to givinostat exhibit a clear pattern of early occurrence. Just over half (50.79%) occur within the first 0–30 days of treatment, which is directly consistent with the main safety signals of the drug, such as thrombocytopenia and gastrointestinal reactions. Therefore, the first 30 days are critical for safety monitoring. During this period, clinicians and pharmacists should conduct regular blood routine and lipid tests and actively manage early gastrointestinal symptoms to ensure patient tolerance and the continuity of medication.

**FIGURE 5:**
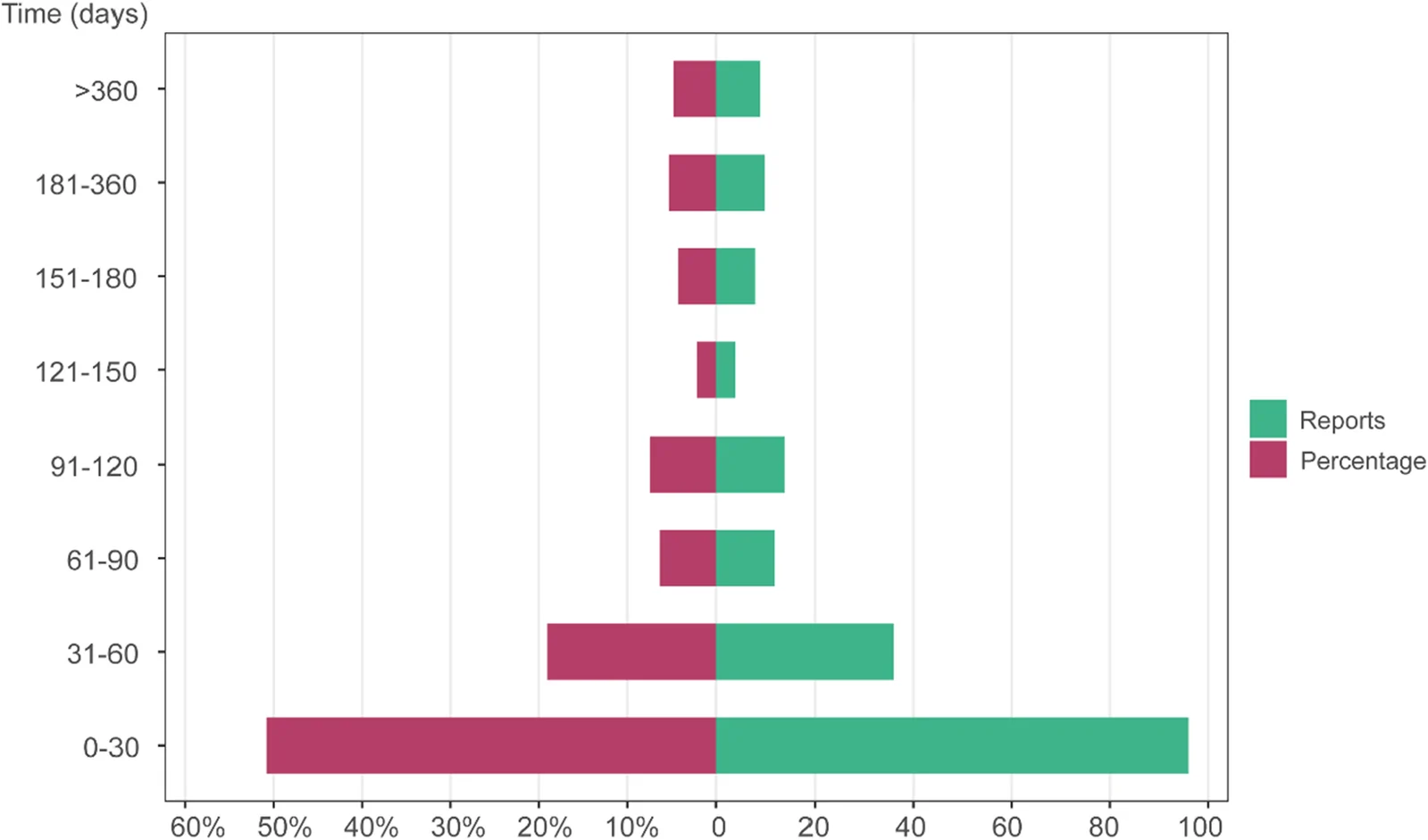
Distribution of time to onset of givinostat-associated adverse events by time interval.

### 3.5 Serious vs. non-serious cases

Among the 321 givinostat-related AE reports, 64 (19.9%) were classified as serious outcomes, including DE, HO, LT, or OT, while 257 (80.1%) were non-serious. To identify potential risk factors for severity and specific AEs prone to serious outcomes, we compared the constituent ratios between the serious and non-serious groups. Only PTs with a total report frequency ≥5 were included in the formal statistical tests, using Chi-square or Fisher’s exact test as appropriate. As shown in Table 5, patients in the serious group had a significantly higher median body weight compared to those in the non-serious group (56.5 kg vs. 44.0 kg, *p* = 0.048), a borderline difference in unadjusted analysis that warrants cautious interpretation. Serious cases showed a non-significant trend toward older age (median 17.5 vs. 14.0 years; *p* = 0.055). However, the estimate was imprecise because of the small number of serious reports.

**TABLE 5:** Exploratory univariate comparison of adverse event profiles between serious and non-serious reports among givinostat users.

Characteristic	Serious	Non-serious	Statistic	*p*-value
Age, years (median, IQR)	17.5 (12–20.75)	14 (11–19)	1.916^d^	0.055^c^
Weight, kg (median, IQR)	56.5 (38.75–78.5)	44 (32–64)	1.98^d^	0.048^c^
Types of AEs, *n* (%)	​	​	​	​
Abdominal discomfort	4 (12.90)	27 (87.10)	3.72^b^	0.054^a^
Blood triglycerides increased	7 (14.89)	40 (85.11)	4.75^b^	0.029^a^
Diarrhoea	15 (24.19)	47 (75.81)	0.83^b^	0.363^a^
Nausea	9 (33.33)	18 (66.67)	0.03^b^	0.870^a^
Platelet count decreased	14 (14.58)	82 (85.42)	11.73^b^	0.001^a^
Pyrexia	7 (63.64)	4 (36.36)	–	0.021^e^
Thrombocytopenia	16 (53.33)	14 (46.67)	6.94^b^	0.008^a^
Vomiting	6 (26.09)	17 (73.91)	0.04^b^	0.848^a^

AEs, were selected for the main table if they showed nominal statistical significance in unadjusted seriousness comparisons or were of high clinical frequency.

^a^ Proportions were compared using Pearson *χ2* test.

^b^ The *χ2* statistic of the Pearson *χ2* test.

^c^ Mann-Whitney U test.

^d^ The Z statistic of the Mann-Whitney U test.

^e^ Fisher’s exact test.

*p* values <0.05 were considered statistically significant. Data are presented as n (%) for categorical variables and median (IQR) for continuous variables. *p* values were derived from Pearson’s *χ2* test, Fisher’s exact test, or Mann-Whitney U test as appropriate. These are unadjusted nominal *p* values for descriptive exploration; confirmatory inference with false discovery rate control is presented in Table 7.

As shown in Table 5, univariate analysis revealed that 4 AEs exhibited statistically significant differences in report distributions between serious and non-serious cases (*p* < 0.05). Specifically, thrombocytopenia (53.33% vs. 46.67%, *p* = 0.008) and pyrexia (63.64% vs. 36.36%, *p* = 0.021) were disproportionately concentrated in the serious group. Conversely, platelet count decreased (14.58% vs. 85.42%, *p* = 0.001), blood triglycerides increased (14.89% vs. 85.11%, *p* = 0.029) were predominantly associated with non-serious reports, demonstrating a significantly lower serious outcome proportion compared to other AEs. Notably, although platelet count decreased was the most frequently reported PT (*n* = 96), its unadjusted proportion of serious outcomes was relatively low (14.58%). In contrast, thrombocytopenia, representing the clinical diagnosis counterpart, demonstrated a higher serious proportion (53.33%), illustrating a preliminary divergence between laboratory abnormalities and clinical diagnoses. Furthermore, no significant differences were observed for common gastrointestinal AEs such as diarrhoea (*p* = 0.363), nausea (*p* = 0.870), or vomiting (*p* = 0.848), indicating that the majority of these events were mild-to-moderate and manageable in the real-world setting. For abdominal discomfort, the difference approached but did not reach statistical significance (*p* = 0.054), and this finding should be considered exploratory/hypothesis-generating. No significant differences were observed for the remaining PTs, and the complete comparison is provided in Supplementary Table S1.

### 3.6 Age, weight, and corticosteroid use as predictors of SAEs in givinostat therapy

To evaluate whether age, weight, and corticosteroid use were independent predictors of serious adverse events (SAEs), we performed univariable and multivariable logistic regression using the dichotomized age and weight groups and the binary corticosteroid use variable, as defined in the Methods section. A total of 185 patients with complete data on age, weight, and corticosteroid use were included in the final analysis, among whom 51 (27.6%) experienced at least one SAE. The proportion of reports with SAE was 23.8% (30/126) in patients aged <18 years *versus* 35.6% (21/59) in those aged ≥18 years, 24.2% (23/95) in patients weighing ≤45 kg *versus* 31.1% (28/90) in those weighing >45 kg, and 23.1% (24/104) in those not receiving corticosteroids *versus* 33.3% (27/81) in those receiving corticosteroids. In univariable analysis, none of the three factors was significantly associated with SAE: age ≥18 years (OR = 1.77, 95% CI 0.90–3.46, *p* = 0.097), weight >45 kg (OR = 1.41, 95% CI 0.74–2.72, *p* = 0.295), and corticosteroid use (OR = 1.67, 95% CI 0.87–3.21, *p* = 0.123). After mutual adjustment in the multivariable model, the associations remained non-significant: age ≥18 years (adjusted OR = 1.64, 95% CI 0.79–3.37, *p* = 0.182), weight >45 kg (adjusted OR = 1.21, 95% CI 0.60–2.45, *p* = 0.588), and corticosteroid use (adjusted OR = 1.66, 95% CI 0.87–3.22, *p* = 0.127). No statistically significant independent association with SAEs was identified for age, weight, or corticosteroid use in this complete-case analysis. However, given the limited sample size and wide confidence intervals, these null findings should be interpreted as exploratory and do not exclude the possibility of modest associations (Table 6).

**TABLE 6:** Univariable and multivariable logistic regression analyses of age, weight and corticosteroid use as risk factors for SAEs in givinostat-treated patients.

Dependent: SAE	Non-serious	Serious	OR (multivariable)	OR (univariable)
Age				
<18	96 (76.2)	30 (23.8)	–	–
≥18	38 (64.4)	21 (35.6)	1.64 (0.79–3.37, *p* = 0.182)	1.77 (0.90–3.46, *p* = 0.097)
Weight				
≤45	72 (75.8)	23 (24.2)	–	–
>45	62 (68.9)	28 (31.1)	1.21 (0.60–2.45, *p* = 0.588)	1.41 (0.74–2.72, *p* = 0.295)
Corticosteroid use				
No	80 (76.9)	24 (23.1)	–	–
Yes	54 (66.7)	27 (33.3)	1.66 (0.87–3.22, *p* = 0.127)	1.67 (0.87–3.21, *p* = 0.123)

### 3.7 Subgroup disproportionality analysis for host factors

The above univariate comparisons (Table 5) identified several AEs with nominally significant differences in seriousness proportions. However, these exploratory findings are subject to multiple testing and do not account for the background AE distribution of givinostat. To address these limitations, we further applied an LRT framework with BH adjustment, which compares each PT against all other PTs within the givinostat reports, thereby providing a confirmatory assessment of host-factor-related reporting bias. The results of this adjusted analysis are summarized in Table 7.

**TABLE 7:** LRT-based subgroup disproportionality analysis for seriousness (Serious vs. Non-serious).

Host factor	PTs evaluated	LRT	adj. *p*	Direction (proportion)
Seriousness	Thrombocytopenia	3.51	0.071	Higher (53.33%)
​	Blood triglycerides increased	2.94	0.151	Lower (14.89%)
​	Platelet count decreased	6.27	0.024	Lower (14.58%)
​	Pyrexia	2.63	0.132	Higher (63.64%)
Age	None	​	–	No bias detected
Weight	None	​	–	No bias detected
TTO	None	​	–	No bias detected

The LRT compares each PT against all other PTs within the givinostat reports. PTs with adj. *p* < 0.05 were considered statistically significant; PTs with adj. *p* ≥ 0.05 are shown for descriptive purposes. Direction compares each PT-specific serious proportion with the overall PT-entry-level serious proportion (30.07%), calculated as the number of PT entries from reports with serious outcomes divided by the total number of PT entries in all givinostat reports. Parenthetical values are PT-specific proportions.

Among the host factors analyzed, platelet count decreased was the only PT that reached statistical significance after BH adjustment, with a significantly lower serious proportion (14.58%) than the overall PT-entry-level serious proportion among all givinostat reports (30.07%, adj. *p* = 0.024). Thrombocytopenia (53.33%, adj. *p* = 0.071), blood triglycerides increased (14.89%, adj. *p* = 0.151), and pyrexia (63.64%, adj. *p* = 0.132) did not reach statistical significance, but these estimates were imprecise because of the small number of reports. Confirmatory studies are needed. Notably, pyrexia showed a numerically higher serious proportion (63.64% vs. 30.07%); however, due to the limited number of reports and the resulting wide confidence interval, this association should be interpreted as a trend rather than a definitive signal. For age (<18 vs. ≥ 18 years), weight (≤45 kg vs. > 45 kg), and TTO (≤30 days vs. > 30 days), no PT reached statistical significance (all adj. *p* > 0.05), indicating that these host factors did not substantially modify the AE reporting profile of givinostat.

To further characterize the temporal pattern of key AEs, we performed a TTO analysis for PTs with significant disproportionality signals (Table 8). Among the 321 givinostat-related reports, 124 (38.6%) had evaluable TTO data. Diarrhoea tended to occur early, with a median TTO of 3.5 days (IQR: 0.25–18.25) for non-serious diarrhoea reports. In contrast, haematological abnormalities showed a more delayed onset: the median TTO for non-serious platelet count decreased was 15.5 days (IQR: 14.25–32.5). In descriptive comparisons, the median TTO for serious reports of platelet count decreased was 63 days *versus* 15.5 days for non-serious reports. However, this comparison is based on very small numbers (*n* = 4 and 6, respectively) and was not adjusted for multiple testing, precluding any firm interpretation. For thrombocytopenia and diarrhoea, median TTO was similar between seriousness groups.

**TABLE 8:** TTO for key AEs by seriousness.

PT	Serious		Non-serious		*p*-value*
​	n	Median TTO, days (IQR)	n	Median TTO, days (IQR)	​
Platelet count decreased	4	63 (54.75–182.00)	6	15.5 (14.25–32.50)	0.014
Thrombocytopenia	4	18 (12.75–29.25)	9	17 (2.00–24.00)	–
Diarrhoea	4	9.5 (3.00–28.50)	14	3.5 (0.25–18.25)	–

* *p* values are shown for descriptive purposes only; they were not adjusted for multiple comparisons and, given the very small sample sizes (*n* < 5 in some groups), should be interpreted as exploratory rather than confirmatory.

### 3.8 Active-comparator analysis

To explore whether the main signals were shared by other DMD therapies, we compared selected PTs across givinostat, prednisone, deflazacort, and vamorolone (Table 9). For several comparators, the number of reports was too small to calculate stable RORs, and these are marked as “–”. Givinostat showed stronger signals for platelet count decreased (ROR 91.70, 95% CI 73.97–113.68) and thrombocytopenia (ROR 23.76, 95% CI 16.48–34.24). Vamorolone showed a positive disproportionality signal for platelet count decreased (ROR 2.39, 95% CI 1.38–4.12) but no positive signal for thrombocytopenia (ROR 0.17, 95% CI 0.02–1.19). For prednisone and deflazacort, RORs for platelet count decreased and thrombocytopenia could not be calculated because of insufficient reports. Prednisone and deflazacort did not show significant signals for platelet count decreased and thrombocytopenia. For diarrhoea, givinostat had an ROR of 8.29 (95% CI 6.39–10.75), whereas deflazacort had an ROR of 0.46 (95% CI 0.06–3.29); data for prednisone and vamorolone were insufficient. These findings are exploratory because of the small number of reports for some comparators and differences in reporting patterns across drugs.

**TABLE 9:** Active-comparator disproportionality analysis of selected adverse event signals across DMD therapies.

PT	Givinostat ROR (95% CI)	Prednisone ROR (95% CI)	Deflazacort ROR (95% CI)	Vamorolone ROR (95% CI)
Thrombocytopenia	23.76 (16.48–34.24)	–	–	0.17 (0.02–1.19)
Platelet count decreased	91.70 (73.97–113.68)	–	–	2.39 (1.38–4.12)
Diarrhoea	8.29 (6.39–10.75)	–	0.46 (0.06–3.29)	​

## 4 Discussion

In this real-world pharmacovigilance study based on 321 adverse event reports from the FAERS database, we systematically characterized the safety profile of givinostat in patients with Duchenne muscular dystrophy (DMD). Our findings confirm that thrombocytopenia and hypertriglyceridemia are the most prominent safety signals, consistent with the known class effects of HDAC inhibitors. More importantly, through a likelihood ratio test (LRT) framework with false discovery rate control, we found that platelet count decreased was the only PT significantly associated with serious outcomes, with a lower serious proportion than the overall baseline. Thrombocytopenia showed a higher serious proportion but did not reach statistical significance after BH adjustment. We first discuss the mechanistic plausibility of the main safety signals and the implications of the LRT-based seriousness findings, then interpret the negative subgroup results, and finally propose practical risk-management strategies based on these observations.

### 4.1 Comparison with existing evidence and mechanistic plausibility

The LRT-based subgroup analysis provided a nuanced refinement of these signals. While thrombocytopenia (clinical diagnosis) had the highest serious proportion in descriptive analyses the LRT identified platelet count decreased as the sole PT with a statistically significant deviation from the overall serious baseline. Notably, the laboratory counterpart ‘platelet count decreased’ had a much lower serious proportion, suggesting that laboratory abnormalities do not always translate to clinically documented serious events, which is a discrepancy that may reflect reporting biases rather than true differences in clinical severity. The clinical rationale for continued use of givinostat despite these hematological risks is further supported by emerging evidence of cardioprotective effects, which is particularly relevant given that cardiomyopathy remains a leading cause of mortality in DMD patients. In preclinical models, givinostat has been shown to restore the physiological microenvironment at the neural–cardiac junction by attenuating macrophage-mediated overrelease of chondroitin sulfate proteoglycan 4 (CSPG4), a key inhibitor of neuronal sprouting associated with dilated cardiomyopathy (Milan et al., 2025). This resulted in reduced cardiac fibrosis and improved cardiac function. Thus, the hematological and metabolic toxicities identified in our study should be weighed against both the established skeletal muscle benefits and the potential cardiac benefits, reinforcing the importance of structured monitoring to enable continued therapy rather than to preclude its use. Finally, to explore whether the main givinostat signals could be attributed to background corticosteroid therapy, we performed an exploratory active-comparator analysis. Platelet-related signals for givinostat were substantially stronger than those for prednisone, deflazacort, or vamorolone. Vamorolone showed a weak signal for platelet count decreased but no signal for thrombocytopenia; data on hypertriglyceridemia were insufficient for comparison. These observations argue against the possibility that the platelet-related givinostat signals are solely due to background corticosteroid therapy. However, the exceptionally high ROR for hypertriglyceridemia (ROR 341.3) requires cautious interpretation. Underlying disease activity and inflammatory burden, as observed in other chronic inflammatory conditions, may confound lipid profiles (Yan et al., 2023). In addition, long-term glucocorticoid therapy can directly affect lipid metabolism through receptor-mediated mechanisms, and glucocorticoid-induced dyslipidemia is dose- and duration-dependent (Huang et al., 2023). Lipid monitoring may also be more frequent in DMD patients than in the general FAERS population, increasing detection and reporting of asymptomatic triglyceride elevations. Moreover, concomitant corticosteroid use was reported in 81 of 185 cases with complete data (43.8%), but the number of hypertriglyceridemia events within strata was too small for stable stratification. Therefore, the hypertriglyceridemia signal cannot be attributed to givinostat alone. It should be regarded as hypothesis-generating and may reflect a combination of drug effects, underlying disease, concomitant glucocorticoids, and surveillance bias. RORs are not incidence rates, and differences in reporting, indication, and patient characteristics preclude direct head-to-head comparisons; these results should therefore be interpreted as supportive rather than definitive.

### 4.2 Impact of missing data on the interpretation of seriousness analyses

OUTC data were missing for 257 of 321 reports (80.1%), and data on route of administration were missing for 91.28% of reports. Since FAERS does not explicitly code non-serious events, the 257 reports lacking DE, HO, LT, or OT were operationally classified as having “no documented serious outcome” rather than as confirmed non-serious events. Unrecorded outcomes may be more likely to be non-serious, as serious events may be preferentially reported with complete outcome fields. However, this assumption cannot be verified in FAERS; missingness may also reflect incomplete data entry, limited follow-up, or reporting from sources with incomplete outcome information. If a substantial proportion of records with missing OUTC data were actually serious, they would be misclassified into the comparison group, biasing comparisons between serious and no-documented-serious outcomes toward the null and reducing statistical power. Conversely, if records with missing OUTC data were predominantly non-serious, the current grouping would approximate the true distribution; however, this remains unproven. Because only 64 reports had documented serious outcomes, all analyses involving seriousness, including univariate comparisons, logistic regression, LRT-based subgroup analysis, and TTO by seriousness, should be considered exploratory and hypothesis-generating. Substantial missingness in route of administration precludes meaningful exposure–response assessment and may introduce residual confounding. Future validation using registries or electronic health records with complete outcome ascertainment is warranted.

### 4.3 Discrepancy between univariate and LRT-based analyses

Although four PTs showed nominally significant differences in serious proportions in the unadjusted analysis (Table 5), only platelet count decreased remained significant after BH-adjusted LRT (adj. *p* = 0.024); thrombocytopenia, blood triglycerides increased, and pyrexia did not. This discrepancy likely reflects multiple-testing correction, the conservative normal approximation of the LRT in sparse data, and limited statistical power. First, the BH procedure controls the false discovery rate and imposes a penalty that is proportional to the number of PTs tested; PTs with modest effect sizes and raw *p* values close to 0.05 are therefore likely to lose significance after adjustment. Second, the LRT approach differs methodologically from the univariate χ^2^ or Fisher’s exact tests: the LRT compares each PT against all other PTs within the same givinostat reports, using a normal approximation, which is more conservative in sparse data settings. Third, the relatively small sample size limited statistical power, particularly for PTs with low event counts such as pyrexia and thrombocytopenia. Consequently, the nominally significant findings in Table 5 should be interpreted as exploratory trends rather than definitive signals. In contrast, platelet count decreased had a sufficiently large effect to withstand BH correction, reinforcing its robustness as the only PT significantly associated with serious outcomes after adjustment. These results highlight the importance of confirmatory analyses with False Discovery Rate (FDR) control when mining spontaneous reporting databases.

### 4.4 Interpretation of negative findings

No significant PT-level bias was detected across age, weight, or TTO subgroups. Regarding the temporal profile, while gastrointestinal events typically manifested within the first week of therapy, the TTO patterns for platelet-related abnormalities differed by seriousness. For non-serious reports of platelet count decreased, the median TTO was 15.5 days, which is broadly consistent with prior pharmacokinetic/pharmacodynamic modeling that predicted a mean 45% platelet reduction with a maximum effect occurring within 28 days of treatment initiation (Fiorentini et al., 2023). This concordance suggests that the peak hematological monitoring window should be centered around weeks 2–4 of therapy. In contrast, the notably later median TTO observed in serious reports of platelet count decreased (63 days) may reflect ascertainment bias, including delayed clinical recognition or reporting, or cumulative dose-related effects, although the very small case numbers (*n* = 4) preclude definitive conclusions. These results should be interpreted alongside our separate logistic regression analysis, in which adult patients showed a non-significant trend toward higher odds of any SAE. Taken together, these complementary findings suggest that adults may have a higher overall SAE risk, likely reflecting the natural history of DMD progression rather than drug-specific toxicity. However, the type of AEs they experience does not differ substantially from that in younger patients. Clinicians should intensify overall monitoring in adult DMD patients but can apply the same AE-specific management protocols across age groups.

### 4.5 Clinical implications and practical recommendations

Based on our findings, we propose the following risk-management strategy for givinostat therapy in DMD patients. Complete blood counts, particularly platelet levels, should be monitored at baseline and weekly during the first month of therapy. Although platelet count decreased exhibited a significantly lower proportion of serious outcomes compared to the baseline (14.58% vs. 30.07%, adj. p = 0.024), its status as the most frequently reported adverse event (29.91%), coupled with a median time-to-onset of 15.5 days, underscores its critical role as an early, highly prevalent indicator. Intensive early hematological monitoring is therefore essential to detect early asymptomatic drops in platelet counts and prevent progression to severe clinical outcomes such as overt thrombocytopenia. Dose interruption or reduction should be considered if platelet count drops below 75 × 10^9^/L, in line with previous HDAC inhibitor guidelines. Lipid profiles should be monitored periodically, as hypertriglyceridemia was the strongest signal by effect magnitude. Although diarrhoea and nausea were frequently reported, their predominantly non-serious nature suggests that symptomatic management, including antiemetics, dietary adjustments, may suffice in most cases without treatment interruption. Adult patients warrant closer overall clinical surveillance for SAEs, but specific AE-type monitoring does not require age-based modification.

## 5 Limitations

Several limitations of this study should be acknowledged. First, FAERS is a spontaneous reporting system subject to under-reporting, over-reporting, and incomplete information. Because FAERS does not explicitly code non-serious events, reports without serious outcome codes were classified as “no documented serious outcome.” This high, likely non-random missingness may introduce misclassification and selection bias and limits statistical power. Therefore, all serious *versus* no-documented-serious comparisons should be regarded as exploratory and hypothesis-generating rather than confirmatory, and independent validation with more complete outcome ascertainment is needed. In addition, the predominance of reports in 2024 (82.6%) aligns with the initial launch of givinostat, which may have inflated early signal estimates and warrants cautious interpretation of temporal trends. Second, the absence of denominator data precludes the calculation of incidence rates. Consequently, ROR and PRR reflect reporting odds rather than clinical risk, and the signal strength for hypertriglyceridemia (ROR 341.3) may partly stem from routine lipid monitoring rather than intrinsic toxicity. Third, the high missing rates for certain variables, particularly the reporting year and route of administration, limited our ability to assess temporal trends and exposure–response relationships. Given that nearly all patients were males with DMD, many receive chronic glucocorticoid therapy, which is a well-established cause of both dyslipidemia and hematological shifts. Fourth, the near-complete male predominance in givinostat reports (96.0%) prevented meaningful sex-based subgroup analyses, which are relevant given the X-linked nature of DMD. Fifth, the relatively small sample size (*n* = 321 AEs) may have limited statistical power for detecting subtle differences in subgroup analyses, and our negative findings should be interpreted with caution. Finally, as with all FAERS-based analyses, the reported associations do not imply causation and should be considered hypothesis-generating rather than confirmatory.

## 6 Conclusion

In this FAERS-based pharmacovigilance analysis, platelet count decreased, thrombocytopenia and hypertriglyceridemia were identified as the most prominent safety signals associated with givinostat in DMD patients. Notably, LRT-based subgroup analysis identified platelet count decreased as the only PT significantly associated with serious outcomes, with a lower serious proportion than the overall baseline (14.58% vs. 30.07%, adj. *p* = 0.024). Thrombocytopenia showed a higher serious proportion (53.33%) but did not reach statistical significance (adj. *p* = 0.071). The consistency of the AE profile across age, weight, and TTO subgroups supports the generalizability of these safety observations. Our findings underscore the importance of structured hematological and metabolic monitoring, particularly in the early treatment phase, and provide a foundation for evidence-based risk-management strategies in givinostat-treated DMD patients.

## Data Availability

The datasets analyzed in this study are publicly available from the FDA FAERS database (https://fis.fda.gov/extensions/FPD-QDE-FAERS/FPD-QDE-FAERS.html). No additional primary data were generated.
