# Supplementary material for: A disproportionality analysis of adverse event reports for givinostat in the FDA adverse event reporting system (FAERS) database
Source: Front Pharmacol. 2026 Oct 21;17:1954821. doi: 10.3389/fphar.2026.1954821 (PMC13731368; doi:10.3389/fphar.2026.1954821)
Supplement: Supplementary file 1 [file DataSheet1.pdf]

Supplementary Material

Supplementary Table S1. Exploratory univariate comparison of adverse event profiles between serious and non-serious reports among givinostat users.

	Serious	Non-Serious	Statistic	*p*-value
Age, years (median, IQR)	17.5 (12–20.75)	14 (11–19)	1.916[d]	0.055[c]
Weight, kg (median, IQR)	56.5 (38.75–78.5)	44 (32–64)	1.98[d]	0.048[c]
Types of AEs, n (%)				
abdominal discomfort	4（12.90)	27(87.10)	3.72[b]	0.054[a]
abdominal pain	3（25)	9(75)	–	1.000[e]
abdominal pain upper	3（23.08)	10(76.92)	–	0.764[e]
alopecia	1（7.69)	12(92.31)	–	0.123[e]
blood triglycerides increased	7（14.89)	40(85.11)	4.75[b]	0.029[a]
contusion	2（14.29)	12(85.71)	–	0.249[e]
decreased appetite	2（33.33)	4(66.67)	–	1.000[e]
diarrhoea	15（24.19)	47(75.81)	0.83[b]	0.363[a]
dizziness	1（20)	4(80)	–	1.000[e]
epistaxis	2（28.57)	5(71.43)	–	1.000[e]
fall	1（20)	4(80)	–	1.000[e]
fatigue	2（33.33)	4(66.67)	–	1.000[e]
gastrointestinal disorder	3（17.65)	14(82.35)	0.74[b]	0.389[a]
headache	3（60)	2(40)	–	0.163[e]
heart rate increased	3（60)	2(40)	–	0.163[e]
illness	2（28.57)	5(71.43)	–	1.000[e]
myalgia	5（45.45)	6(54.55)	–	0.321[e]
nausea	9（33.33)	18(66.67)	0.03[b]	0.870[a]
petechiae	2（40)	3(60)	–	0.640[e]
platelet count decreased	14（14.58)	82(85.42)	11.73[b]	0.001[a]
pyrexia	7（63.64)	4(36.36)	–	0.021[e]
rash	1（16.67)	5(83.33)	–	0.674[e]
thrombocytopenia	16（53.33)	14(46.67)	6.94[b]	0.008[a]
vomiting	6（26.09)	17(73.91)	0.04[b]	0.848[a]

The AEs listed above were AEs with significant signal strengths

[a]Proportions were compared using Pearson χ^2 test.

[b]The χ^2 statistic of the Pearson χ^2 test.

[c]Mann-Whitney U test.

[d]The Z statistic of the Mann-Whitney U test.

[e]Fisher's exact test

p-value < 0.05 were considered statistically significant.

Notes: Data are presented as n (%) for categorical variables and median (IQR) for continuous variables. *p* values were derived from Pearson's χ^2 test, Fisher's exact test, or Mann-Whitney U test as appropriate.
